# Cell-associated HIV RNA: a dynamic biomarker of viral persistence

**DOI:** 10.1186/1742-4690-10-41

**Published:** 2013-04-15

**Authors:** Alexander O Pasternak, Vladimir V Lukashov, Ben Berkhout

**Affiliations:** 1 Department of Medical Microbiology, Laboratory of Experimental Virology, Center for Infection and Immunity Amsterdam (CINIMA), Academic Medical Center, University of Amsterdam, Meibergdreef 15, Amsterdam, 1105 AZ, The Netherlands

**Keywords:** Cell-associated HIV RNA, Virological biomarker, HIV-1 persistence, HIV-1 reservoir, Antiretroviral therapy, HIV cure

## Abstract

In most HIV-infected individuals adherent to modern antiretroviral therapy (ART), plasma viremia stays undetectable by clinical assays and therefore, additional virological markers for monitoring and predicting therapy responses and for measuring the degree of HIV persistence in patients on ART should be identified. For the above purposes, quantitation of cell-associated HIV biomarkers could provide a useful alternative to measurements of viral RNA in plasma. This review concentrates on cell-associated (CA) HIV RNA with the emphasis on its use as a virological biomarker. We discuss the significance of CA HIV RNA as a prognostic marker of disease progression in untreated patients and as an indicator of residual virus replication and the size of the dynamic viral reservoir in ART-treated patients. Potential value of this biomarker for monitoring the response to ART and to novel HIV eradication therapies is highlighted.

## Review

### Introduction

The concentration of free virus particles in blood plasma (plasma viremia or plasma viral load), represented by the copy number of virion RNA per milliliter of plasma, which can be reliably quantified by PCR methods, is traditionally used as the biomarker of HIV-1 replication [1-3]. The main goal of antiretroviral therapy (ART) is suppression of plasma viremia to below the detection limit of the most sensitive assay available in the clinic, and maintaining this “undetectability”. In most HIV-infected individuals treated with modern ART regimens and adherent to therapy, this goal is achieved and therefore, additional virological markers for monitoring the response to therapy should be identified. Ideally, such markers should be predictive of future ART complications (e.g. therapy failure due to suboptimal adherence) and should indicate the necessity of a clinical or behavioral intervention. In addition, recent efforts towards a functional or sterilizing HIV cure [4] have promoted a renewed interest in the development of quantitative and sensitive assays for HIV biomarkers, which should allow the precise measurement of HIV reservoirs and aid in monitoring the effectiveness of the novel therapies aimed at eliminating or reducing these reservoirs [5-8].

**Figure 1:**
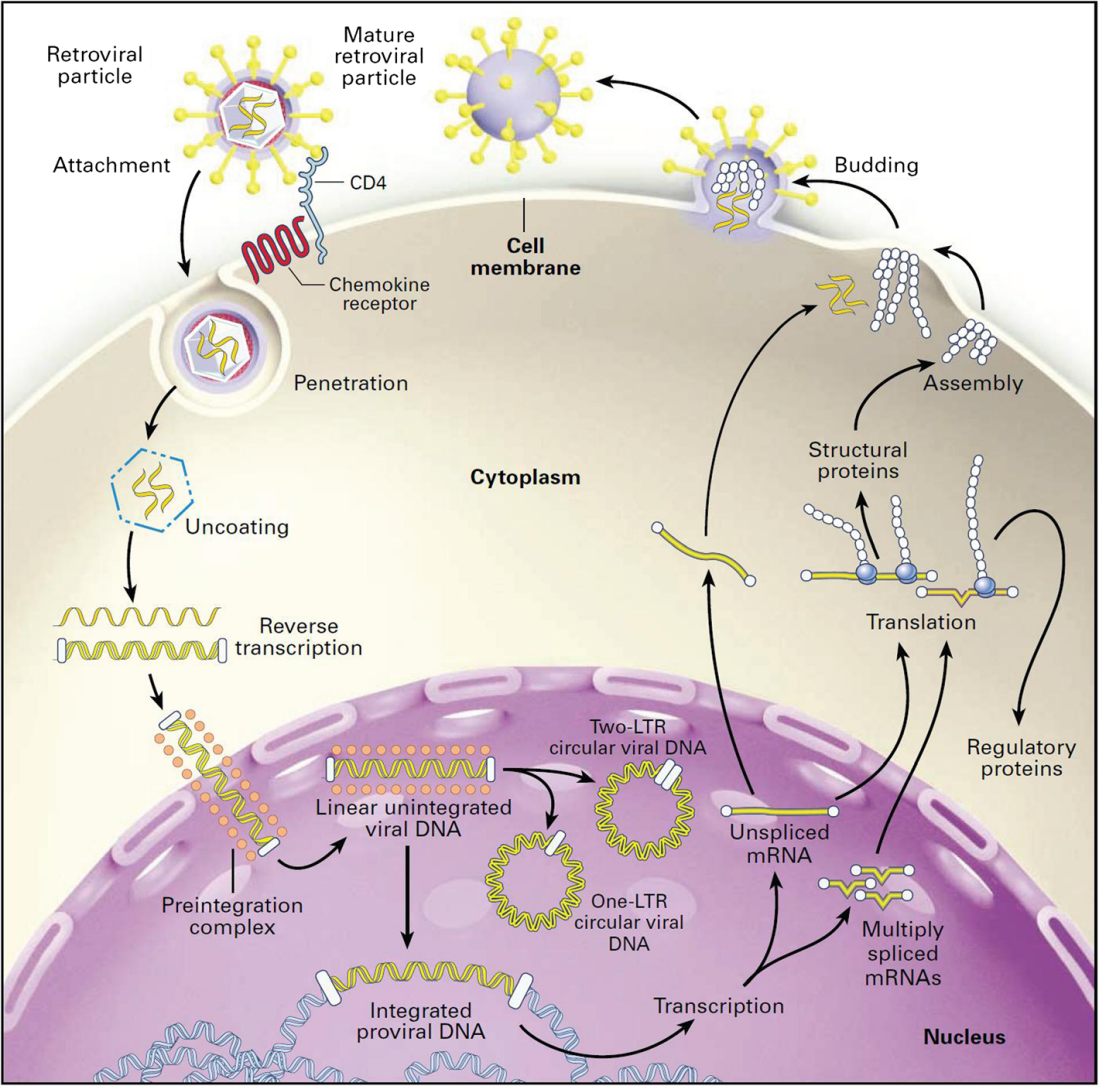
**The essential steps in the life cycle of HIV-1.** The first step is the attachment of the virus particle to receptors on the cell surface. The HIV-1 RNA genome then enters the cytoplasm as part of a nucleoprotein complex. The viral RNA genome is reverse-transcribed into a DNA duplex, which has terminal duplications known as long terminal repeats (LTRs). The linear viral DNA molecule is part of the preintegration complex that enters the nucleus. In the nucleus, unintegrated viral DNA is found in both linear and circular forms. The unintegrated circular forms of viral DNA have either one or two LTRs, are byproducts of the integration process, and are found exclusively in the nucleus. The linear unintegrated viral DNA is the precursor of integrated proviral DNA, which is a stable structure that remains indefinitely in the host-cell genome and serves as a template for viral transcription. Transcription of the proviral DNA template and alternative RNA splicing creates spliced viral RNA species encoding the viral accessory proteins, including Tat, Rev, and Nef, and the unspliced viral RNA encoding the viral structural proteins, including the Gag–Pol precursor protein. All the viral transcripts are exported into the cytoplasm, where translation and assembly and processing of the retroviral particle take place. The cycle is completed by the release of infectious retroviral particles from the cell. (Figure adapted from [12]; reproduced, with permission, from Massachusetts Medical Society © 1999).

It seems logical that, for the above purposes, quantitation of cell-associated (CA) HIV biomarkers could provide a useful alternative to the measurements of plasma viremia. Several viral nucleic acid forms, including different RNA (e.g. spliced, unspliced) and DNA (e.g. total, integrated, unintegrated) molecules, are present in the infected cell at different points of the lentiviral replication cycle (Figure 1). Because these molecules represent HIV reservoirs with different properties, each of these molecules can, in principle, be utilized as an HIV biomarker using sensitive PCR-based techniques. However, with the exception of an assay for total CA HIV DNA, used for HIV diagnostics in infants [9], no quantitative assay for a CA HIV marker is currently in the clinical practice, despite a large body of research on the topic. Several recent reviews focused on the different molecular forms of CA HIV DNA, such as integrated and unintegrated DNA forms [10,11]. This review concentrates on the CA HIV RNA with an emphasis on its use as a virological biomarker.

### CA HIV RNA in the viral replication cycle

**Figure 2:**
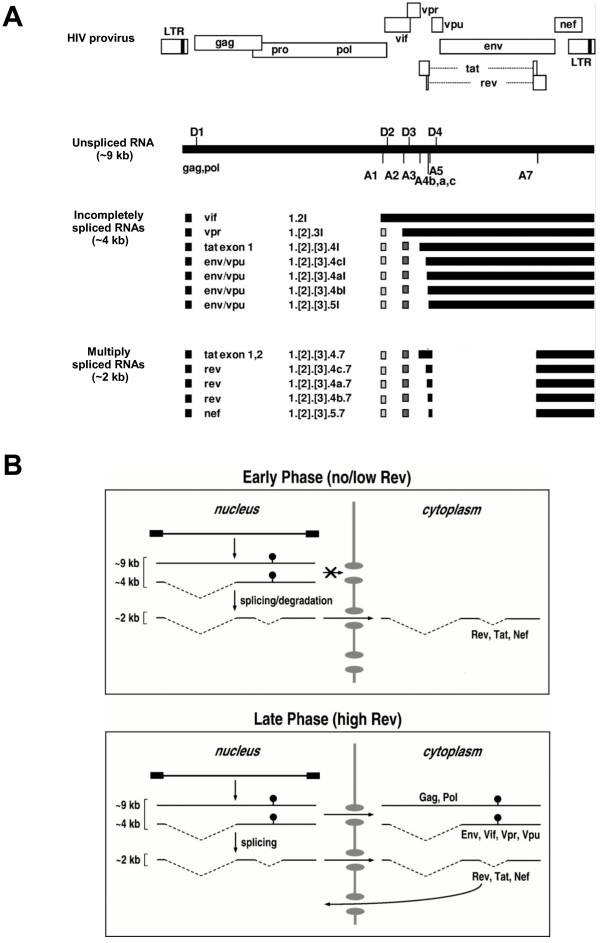
**Viral RNA species produced within HIV-1-infected cells and phases of HIV-1 RNA expression.** (**A**) Viral RNA species produced within HIV-1-infected cells. HIV-1 genes are shown relative to the long terminal repeats (LTR). The viral genomic or 9-kb unspliced RNA shows the location of 5^′^ (D) and 3^′^ (A) splice sites. The incompletely and multiply spliced HIV-1 viral RNAs (4-kb and 2-kb size classes) are shown as black boxes. Spliced RNAs are denoted by the translated open reading frames and by the exon content. (Figure is adapted from [20]; reproduced, with permission, from American Society for Microbiology © 2008.) (**B**) Early and late phases of HIV-1 RNA expression. Full-length unspliced 9-kb RNA, incompletely spliced 4-kb RNA, and multiply spliced 2-kb RNA species are constitutively expressed in the nucleus. In the absence of Rev (upper panel), or when the concentration of Rev is below the threshold necessary for function, the 9-kb and 4-kb transcripts are excluded from the cytoplasm and either spliced or degraded. In contrast, the fully processed 2-kb RNA are constitutively exported to the cytoplasm and used to express Rev, Tat, and Nef. When the levels of Rev in the nucleus are sufficiently high (lower panel), the nuclear export of 9-kb and 4-kb RNAs is activated and the translation of all viral proteins ensues. (Ball and stick) The Rev response element. (Figure adapted from [21]; reproduced, with permission, from Annual Review of Microbiology © 1998).

More than 40 different viral RNAs are produced in HIV-infected cells by alternative splicing of the primary transcript, which is transcribed from the integrated provirus (Figure 2A) [13,14]. Initially, only short (~2 kb) completely spliced, also termed multiply spliced (ms), transcripts are produced, encoding the regulatory proteins Tat, Rev, and Nef. As the infection progresses, there is a shift towards production of ~9 kb unspliced (us) and ~4 kb incompletely spliced (is) transcripts, encoding the structural and accessory proteins Gag, Pol, Env, Vif, Vpr, and Vpu [15-17]. This shift is dependent on the threshold level of the Rev protein, which facilitates the export of the usRNA and isRNA molecules from the nucleus by binding to the RRE (Rev-responsive element), an elongated stem-loop structure located in the Env open reading frame [18,19] (Figure 2B). In addition to its use as a template for translation of the Gag protein and the Gag-Pol polyprotein, usRNA is packaged into progeny viruses as genomic RNA.

### CA HIV RNA in untreated patients

During the 1990s, several groups reported detection and quantitation of CA (us and ms) HIV-1 RNA in peripheral blood cells [22-30] and tissues [31-35] of infected individuals. In these initial studies, qualitative, semiquantitative, or quantitative competitive (QC) reverse transcription (RT)-PCR methods, as well as in situ hybridization-based methods, were used. Before the introduction of combination ART (1996–1997), the main focus of these studies was to verify whether CA HIV RNA could be used as a virological biomarker of disease progression in untreated individuals. Almost 20 years ago, by comparing CA HIV burden (DNA and RNA) between lymphoid tissue mononuclear cells and peripheral blood mononuclear cells (PBMC), it was convincingly demonstrated that during the asymptomatic phase of infection (“clinical latency”) most HIV replication occurs in lymphoid tissues [33]. Nevertheless, a single measurement of HIV-1 msRNA in PBMC in the asymptomatic phase was strongly associated with progression to AIDS in a cohort of 150 homosexual men [36], showing that HIV replication is adequately represented in peripheral blood. Similar predictive power for disease progression was demonstrated for plasma viremia [37,38]. However, time trends of plasma viremia in the asymptomatic phase were found to be highly variable between patients and between studies: a steady-state pattern is usually observed [39], but a U-shaped curve [40] or a gradual increase over time [41] have also been reported. In contrast, CA HIV RNA level in typical progressors was demonstrated by several studies to significantly increase during this phase of infection and to inversely correlate with the CD4^+^ T cell count [23,28,42-44]. Slow progressors, in comparison, typically have lower and relatively constant CA RNA levels [28,42,43,45,46].

We performed a direct comparison of the longitudinal trends of viral RNA in PBMC and plasma (CA HIV RNA in this study was measured by seminested real-time PCR) in a cohort of HIV-infected untreated individuals with a mean follow-up of 55 months [44]. This comparison revealed remarkable differences in the dynamics of these molecular markers. While levels of viral RNA in plasma were stable, those of CA usRNA in PBMC were significantly increasing over time, and levels of usRNA, but not plasma viremia, inversely correlated with the CD4^+^ T cell count. Interestingly, levels of usRNA increased in time significantly faster than those of CA HIV DNA, with a concominant increase of the RNA/DNA ratio [44]. Whether this increase of viral RNA expression in PBMC reflects an increase in the relative numbers of HIV-producing cells per HIV DNA^+^ cell as the infection progresses, or an upregulation of viral transcription in PBMC at the cellular level, remains unclear. Previously, by quantitative microculture assay, Gupta et al. [28] demonstrated that the relative number of HIV-producing cells in untreated individuals parallels the level of usRNA and inversely correlates with the CD4^+^ counts. Progressive weakening of the antiviral immune response during the asymptomatic phase might be one of the factors defining the temporal increase in the relative numbers of HIV-producing cells and therefore the increase in HIV-1 replication rates in PBMC that we and others have described.

Another interesting (and controversial) issue is whether the relative dynamics of usRNA and msRNA in infected individuals is predictive of disease progression. Most viral RNA expressed early by the infected cell is multiply spliced, with unspliced and incompletely spliced RNA forms “taking over” later. Thus, the us/ms RNA ratio in cells from infected individuals can be used as a surrogate marker of the relative numbers of infected cells in the early vs. late stages of virus replication. Several groups [24,42,46], but not all [43,45], reported an increase of the us/ms RNA ratio in PBMC from HIV-infected persons as the infection progresses, and a preponderance of msRNA was demonstrated in long-term nonprogressors compared with typical progressors [47]. In our cohort, a nonsignificant trend towards a longitudinal increase of the us/ms RNA ratio in typical progressors was observed [44].

To explain this phenomenon, the concept of “blocked early-stage latency” has been put forward, by analogy to certain latently infected cell lines (U1, ACH-2) that also demonstrate a preponderance of msRNA production when unstimulated [48,49]. The existence of a significant reservoir of such latently infected cells during the asymptomatic phase of infection in infected individuals was proposed [24]. Alternatively, the difference in the us/ms RNA ratio could be explained by temporal changes in the cytotoxic T lymphocyte (CTL) response. Progressive weakening of the CTL response as the infection progresses would mean that cells in the late, productive phase of infection (which is characterized by an excess of usRNA and isRNA species) are killed at a reduced rate so that more and more cells with an excess of usRNA are present [50]. In agreement with this idea, rapid progression was shown to be associated both with weaker CTL responses and higher us/ms RNA ratios [51].

### CA HIV RNA in ART-treated patients

#### Assays to quantify CA RNA in patients on ART

Already in the early days of combination ART, numerous papers reported detection and quantitation of CA HIV RNA in peripheral blood and tissues of patients on suppressive therapy [12,52-63]. The advent of combination ART roughly coincided with the introduction of real-time PCR methods for nucleic acid quantitation. As a result, most of the developed quantitative assays for CA HIV RNA and DNA in patients on ART are based on real-time PCR [58,64-68]. These assays are highly specific, but to be able to measure minute amounts of HIV nucleic acids in limited biological material from patients on suppressive ART, the assay sensitivity had to be boosted. We achieved this by addition of a limited-cycle (in order to stay in the linear range) preamplification step with seminested primers before the real-time PCR, resulting in a dramatic improvement of assay sensitivity without compromising the linearity of the standard curve [69]. As a result, this seminested real-time PCR assay allows routine detection and quantitation of HIV RNA and DNA in the vast majority of PBMC samples of patients on combination ART with undetectable plasma viremia [69,70]. Notably, this technology results in a dramatic reduction of the necessary amount of input biological material. Other groups have adapted the commercial quantitative tests for HIV-1 plasma viremia (e.g. Roche Amplicor) for the quantitation of CA HIV RNA [55,71,72]. Detection/quantitation of CA HIV RNA in patients on ART by the in situ hybridization-based assays [61,73], as well as by transcription-mediated amplification (TMA) [74] have also been reported.

In any assay, the quantified amount of CA HIV RNA needs to be normalized to the cellular input. However, simple cell counting or total RNA measurement in the sample is insufficient, as differences in efficiencies of both RNA isolation and reverse transcription between different samples also need to be taken into account. We routinely determine the cellular inputs of our cDNA samples by quantifying 18S ribosomal RNA in a separate real-time PCR [69,70]. Housekeeping genes (*e.g.* GAPDH) have also been used for this purpose [66], but there is evidence that 18S ribosomal RNA may be a better choice for normalizing real-time PCR data, especially for virus-infected cells [75]. If one is working with total PBMC, it also makes sense to normalize the CA HIV RNA signal to the percentage of CD4^+^ T cells in the sample. The latter can be determined either directly by flow cytometry or approximated by the CD4^+^ cell count per microliter of blood (which is usually available), as a strong positive correlation is commonly present between CD4^+^ counts and CD4^+^ percentages [76].

One well-known problem that relates to all hybridization-based methods of HIV nucleic acid quantitation is the extreme heterogeneity of HIV sequences, in particular when different virus subtypes are encountered. For real-time PCR methods, this translates into possible effects of mismatches between the primer or probe with their binding sites on the efficiency of real-time PCR, as presence of even a single mismatch may reduce the PCR efficiency by several logs [77-81]. This complicates the analysis, especially when comparing samples of different patients. Using degenerate primers [82] and targeting conserved regions of the HIV genome, like gag, pol, or LTR sequences [83,84], helps to reduce this problem to some extent. Interestingly, two more radical solutions have also been described. One solution is to use patient-matched PCR primers (and probes) for the real-time PCR [67,85]. However, this approach can become quite laborious and expensive if samples from a large number of patients are being studied, as not only the primers and probes, but also the quantitation standards have to be patient-matched and tested. Another shortcoming of this approach is that the matching is done to only one (or possibly several, if degenerate primers are used) predominant viral sequence(s), with the risk of misrepresentation of all minority HIV-1 variants.

We proposed another radical solution: to calculate, for each patient, individual mismatch-related quantification errors (MRQE) and normalize all quantified amounts of HIV RNA (or DNA) to the MRQE values [70]. The MRQE values are produced by performing a real-time PCR in which the patient-derived PCR amplicons, containing the primer and probe target sites, are used as templates. The concentrations of the template amplicons are determined spectrophotometrically and equalized by dilution before real-time PCR. A control template without mismatches is amplified as well. Patient-specific MRQE are calculated as the differences between the log_10_-transformed output copy numbers of the individual patient-derived templates and the control template.

#### Decay kinetics of cell-free and CA HIV RNA upon ART initiation

**Figure 3:**
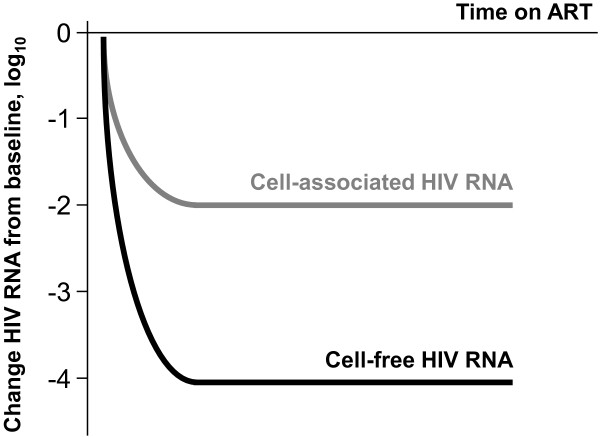
Decay kinetics of cell-free and cell-associated HIV RNA upon ART initiation.

Initiation of combination ART causes a rapid decline in plasma viremia, which occurs in several phases, and almost invariably leads to a level that is undetectable by current commercial assays (20–50 copies/ml). However, by sensitive assays [86-91], low levels of free virus can still be detected in a majority of patients on ART [92]. After several years of therapy, this residual viremia reaches a plateau of 1–10 copies/ml and does not appear to decline any further [93]. The total drop in plasma viremia on ART, depending on pre-therapy values, is thus 3–6 log_10_. Remarkably, CA HIV RNA in PBMC and lymph nodes follows similar decay kinetics upon ART initiation, with a rapid initial decline towards a plateau, but the drop is only 1–2 log_10_, as shown by several groups (Figure 3) [12,59,70,94-98]. Plasma viremia reflects a balance between virus production and virus clearance, and because clearance of free virus in HIV infection was shown to be very rapid, with a half-life in the order of minutes to hours [99,100], plasma viremia is considered to be directly proportional to virus production. Likewise, the viral decay kinetics in plasma upon ART initiation is considered to mimic the decay kinetics of HIV-producing cells [2,101]. In a productively infected cell population, the CA HIV RNA load is also considered to be proportional to virus production. Therefore, in view of classical models of viral dynamics [102], the observed disproportion between the cell-free and CA viral RNA in untreated patients and patients on ART remains puzzling.

To explain this discrepancy, one may assume the existence of a subpopulation of cells that contain viral RNA but either do not produce detectable virus at all, or produce only minute amounts of viral particles. One possibility is that these are latently infected cells that do not produce virus particles due to either insufficient viral RNA transcription, nuclear retention of usRNA or msRNA, or both [64,103]. Obviously, as ART only stops infection of new cells but does not prevent viral RNA transcription, these cells are not expected to decay upon ART initiation and are responsible for the “background level” of CA RNA in patients on ART. Alternatively, this “background level” of CA RNA without a corresponding level of free virus in plasma may reflect residual HIV replication occurring by cell-to-cell transfer, as no detectable free virus is produced during this process (see below).

Another possible explanation for the observed disproportion between the cell-free and CA viral RNA at high and low viral loads is that virion clearance may not be a simple first-order reaction and its rate may be inversely proportional to the viral load, as shown for hepatitis B virus [104]. Interestingly, the clearance rate of SIV in experimentally infused naïve monkeys or infected monkeys with low viral loads was demonstrated to be ~10-fold higher than the HIV clearance rate in untreated chronically infected patients as measured by plasma apheresis [100,105]. Notably, in the latter study, the longest half-life of free virus was measured in the patient whose viral load was ~1 log_10_ higher compared to other patients [100]. In rhesus macaques, neutralizing antibodies (Nab) were shown to accelerate clearance of free virions [106]. The Nab-mediated clearance rate may be saturated at high viral load and especially efficient at low viral load, in particular because HIV-specific Nab titers decline by only ~1 log_10_ on ART, much less than does free virus [107,108], and therefore, more Nab per viral particle would be available at low viral loads. Therefore, virion clearance may be extremely rapid in patients on ART and consequently, less free virus particles may be detected per virus-producing cell in these patients than in untreated patients with high viral loads.

#### Cellular origin of CA HIV RNA in patients on ART

A number of important insights into the persistence of CA HIV RNA in patients on ART have been obtained from the work of Fischer and colleagues [59,67,85,109-112]. In particular, by freeze-thaw nuclease digestion prior to RNA isolation and RT-PCR, they could differentiate between genuine intracellular HIV-1 usRNA and extracellular virion RNA attached to a cell, and demonstrated that the latter represents ~12% of total PBMC-associated usRNA in untreated individuals but only <0,5% in patients on suppressive ART [109]. This means that >99,5% of CA usRNA in patients on ART represents intracellular transcripts. The cellular origin of these transcripts is an important issue in HIV persistence under therapy: these transcripts may originate either (i) from latently infected cells (latency is defined here as lack of virion production), (ii) from cells reactivated from latency to undergo productive infection with virion release but without infection of new cells, or (iii) from cells newly infected despite ART.

Latently infected resting memory CD4^+^ T cells are considered a major HIV reservoir in patients on ART and a main barrier to virus eradication, due to the relative stability of this cell population [113]. By isolating extremely pure populations of resting CD4^+^ T cells from patients on ART, Siliciano’s group demonstrated very low copy numbers of full-length usRNA and msRNA in these cells (<10 copies/10^6^ cells) and an excess of short abortive transcripts that terminate prior to nucleotide 181 [114,115]. In addition, msRNA in resting CD4^+^ T cells from patients on ART was shown to be retained in the nucleus, precluding translation of viral Tat and Rev proteins and consequently high-level transcription and nuclear export of usRNA and isRNA [103]. These effects likely contribute to the latent state of HIV in these cells. Interestingly, the same pattern of nuclear localization of msRNA was recently observed in a chemokine (CCL19) induced model of HIV latency in primary resting CD4^+^ T cells [116]. *In vivo*, a resting memory CD4^+^ T cell can undergo reactivation as a response to antigens or cytokine induction. If this cell is latently infected with HIV, reactivation may trigger the transition to productive infection [117]. In accordance with this, *ex vivo* and *in vitro* stimulation of resting CD4^+^ T cells resulted in relocalization of HIV msRNA to the cytoplasm [103,116].

By PBMC fractionation coupled to limited dilution analysis (LDA) and real-time PCR, Fischer’s group has demonstrated significantly higher per-cell CA HIV RNA load in activated than in resting HIV RNA^+^ CD4^+^ T cells from patients on ART [67]. However, very few cells among the total bulk of HIV DNA^+^ resting cells can be reactivated to productive infection [114], and it therefore remains unclear whether latently or productively infected CD4^+^ T cells are responsible for the largest fraction of CA HIV RNA in patients on ART. Fischer et al. have further shown that >90% of all PBMC-associated HIV RNA in patients on ART is derived from cells that contain low to intermediate amounts of usRNA and low or undetectable amounts of msRNA [112], suggesting that a substantial fraction of these cells is latently infected. A similar conclusion can be drawn from the analysis, by the same authors, of CA HIV RNA in lymphoid tissues of patients on ART [111]. These results seem to contradict the earlier work of Hockett et al. that estimated, using in situ hybridization (ISH) and quantitative PCR methods, that the vast majority of CA HIV RNA in lymphoid tissue of patients on suppressive ART is contained within a few cells with an RNA copy number per cell similar to that in untreated patients (>3 log_10_ copies/cell) [57], suggesting that these cells are productively infected. This apparent contradiction can be attributed to relatively low sensitivity of the ISH method used by Hockett et al., which could have hampered the detection of cells harboring low levels of HIV RNA. Importantly, due to the low patient numbers in both studies, the studies could have selected patients belonging to different populations, e.g. with respect to the CTL responses (see below).

#### CA HIV RNA as a marker of the “active viral reservoir”

The vast majority of cells harboring HIV provirus do not transcribe any viral RNA, both in untreated and ART-treated patients [112]. Some of these cells are able to support productive HIV infection upon reactivation, but unless such cell is reactivated to productive infection, it stays invisible to the immune system as it does not produce any viral antigens. Total CA HIV DNA is therefore a biomarker of the total proviral reservoir, comprised of cells that do or do not transcribe viral RNA. CA HIV RNA, on the other hand, is a biomarker of a subset of the total viral reservoir, containing cells in which HIV sequences are actively transcribed (“active HIV reservoir”). This “active reservoir” should not be seen as a “frozen” population of infected cells with distinct properties, but rather as a “snapshot” of a dynamic system at a particular point in time. As discussed above, a subset of this “active reservoir” may consist of cells in which viral proteins are not produced due to insufficient transcription levels and/or mislocalization of viral RNA. However, a recent report demonstrated that resting CD4^+^ T cells are capable of producing Gag protein without spreading infection in an *in vitro* latency model [118], suggesting another level of HIV latency regulation. If most of the resting CD4^+^ T cells that transcribe some viral RNA can produce some viral proteins, it means that the “active viral reservoir”, for a large part, consists of cells that present antigenic stimulation to the host immune system. In line with this, the presence of CA HIV RNA in patients on ART was shown to directly correlate with lymphoproliferative responses to the HIV-1 p24 antigen [61].

Interestingly, a recent study on elite and “secondary” controllers (patients who control viremia at <50 copies/ml either without treatment or after treatment discontinuation, respectively) also indirectly confirms this notion, as both patient groups had significantly lower usRNA levels as compared to patients on ART with plasma viremia suppressed to <50 copies/ml [119]. In addition, both elite and secondary controllers showed higher T-cell proliferative responses to Gag and Pol peptides [119]. This suggests that natural HIV control, as opposed to the ART-mediated control, may be exerted mainly through host CTL responses that eliminate cells expressing viral antigens (“active HIV reservoir”), possibly explaining why this reservoir in natural controllers was very limited. This is supported by a recent study that found that elite controllers harbor lower levels of integrated HIV DNA than patients on ART, despite comparable levels of total DNA [120]. As transcription/translation from HIV integrated DNA is much more efficient than from unintegrated DNA [11], cells harboring integrated viral DNA and transcribing viral RNA could be preferentially destroyed by the CTL response. Interestingly, the size of the “activatable” HIV reservoir, estimated by the infectious unit per million (IUPM) assay (this assay measures the frequency of infected cells capable of producing virus upon *ex vivo* stimulation), was also shown to be at least 1 log_10_ lower in natural controllers than in patients on ART [121]. In another report, patients who initiated ART early (3–15 weeks after infection) were shown to have up to 2 log_10_ lower usRNA levels and 1 log_10_ lower viral transcription rates (HIV RNA/DNA ratios) under ART than patients that started therapy during chronic infection [85]. This extends the findings of Strain et al. that the replication competent viral reservoir is significantly smaller in subjects starting ART early in infection [122]. Early ART is thought to preserve immune functions and limit the possibilities for HIV to escape from host immunity, likely explaining why a significantly larger fraction of the “active reservoir” was eliminated in patients starting therapy early.

Therefore, in patients on ART, it is plausible that continuous transcription of CA viral RNA and expression of HIV antigens on the surface of infected cells, even in the absence of virion production and/or infection of new cells, would exert continuous pressure on the immune system and cause additional morbidity as a result of persistent immune activation, inflammation, and immunosenescence [123]. Despite the fact that ART has dramatically increased the median survival time of HIV-infected individuals, several studies have found excess mortality rates in the infected and ART-treated population compared to the general population [124-126]. It is unclear whether this excess mortality is due to the effects of HIV infection itself, the adverse effects of the antiretroviral drugs, or to any possible co-infections and co-morbidities. If the former is true, it is necessary to develop quantitative virological biomarkers to monitor these effects. Further studies are warranted to establish whether CA HIV-1 RNA can be used as a reliable surrogate marker of such effects, but several reports already point to a direct correlation of CA HIV RNA levels with markers of immune activation in untreated patients, natural controllers, and patients on ART [127-129].

#### CA HIV RNA as a marker of residual virus replication

Since the introduction of potent ART, it has been unclear whether low-level HIV replication is occurring in (some) patients on ART, and a considerable debate on this matter has been ongoing for some time [7,117,130-135]. Obviously, understanding whether viral reservoirs in patients on ART can be replenished by residual HIV replication is important for designing possible therapeutic interventions (e.g. therapy intensification to abolish the residual replication). In addition, because long-lived latent HIV reservoirs pose a major obstacle to an HIV cure, a number of strategies to eliminate latently infected cells by induction of virus production are currently being tested [5-7]. Notably, complete inhibition of HIV replication seems an absolute prerequisite for any such strategy to work, because if infection of new cells is not completely inhibited, such a strategy may result in further dissemination of the HIV reservoir, instead of virus eradication [4,136].

Direct demonstration of infection of new cells in a patient on ART is, however, extremely difficult, and therefore, a number of studies have attempted to show residual HIV replication by demonstrating virus evolution in patients on ART. However, these studies could not detect virus evolution or emerging drug-resistance mutations in the majority of patients [132,134,135,137-139]. Very recently, data have been reported that suggest that even if some virus evolution is detected in a patient on ART, the evolutionary rate is very low [140]. This lack of significant virus evolution in patients on ART is currently seen as one of the strongest points against residual HIV replication.

Attempts have also been made to demonstrate HIV replication despite therapy by studying levels and longitudinal trends of virological biomarkers. In particular, since the first reports describing persistence of CA HIV RNA in patients on ART, it has been proposed as a possible biomarker of residual virus replication [53,56,58,61]. However, it soon became clear that the mere presence of viral RNA in infected cells does not at all imply virus replication, as CA HIV RNA can be derived from cells reactivated from latency and even from latently infected cells. This is likely true for all CA HIV RNA species, including msRNA. MsRNA production, relative to that of other viral RNA species, is elevated at the early stages of HIV life cycle, and most msRNA in untreated individuals is thought to be derived from newly infected cells [141]. In accordance with this, the decay of msRNA upon ART initiation is much faster than that of usRNA [66,85,94,141], and most studies have reported lower detectability and levels of msRNA compared to usRNA in patients on suppressive ART [67,70,109,111]. Still, the presence of msRNA in patients on ART does not per se signify a recent infection, or even a productive infection. A temporal shift towards a higher msRNA/usRNA ratio in a patient on ART could, however, suggest some new infection events, and therefore, the relative abundance of different CA HIV-1 RNA species is a potentially informative biomarker of residual replication in patients on ART. Such cases have not yet been described, probably due to the extremely low msRNA levels in patients on ART.

UsRNA is more abundant and therefore more easily detected, and several studies could link its expression to the residual virus replication. Two studies that followed patients from the start of ART have reported a reverse correlation between HIV usRNA levels and decay rates of CA HIV DNA on therapy, suggesting that the viral reservoir may have been replenished by ongoing residual replication [66,97]. Some (but not all) groups have measured a substantial reservoir of CA HIV RNA and DNA in monocytes from patients on ART [63,67,142]. As monocytes only circulate in peripheral blood for 1 to 3 days before entering tissues and differentiating into macrophages, the mere presence of HIV infection in these cells was interpreted as evidence for recent infection and residual replication under ART [63].

Another approach to demonstrate residual virus replication under therapy is to show a change in level of a virological biomarker upon an increase (e.g. therapy intensification) or a decrease (e.g. suboptimal adherence) in therapy pressure. A recent study that used such an approach reported a decrease in the level of HIV-1 usRNA in the ileum upon therapy intensification with raltegravir (an integrase inhibitor) [143], suggesting that residual replication may be ongoing in some compartments. Alternatively, residual virus replication can be demonstrated by showing a link between an expression level of a virological biomarker and a certain clinical endpoint. Using this approach, we have demonstrated, by seminested real-time PCR, that higher levels of HIV-1 usRNA in PBMC are predictive of future therapy failure in patients on ART with undetectable plasma viremia [70]. The predictive value of usRNA for virological response to ART was recently confirmed by an independent study that used a completely different RNA detection assay, namely simultaneous ultrasensitive subpopulation staining/hybridization *in situ* (SUSHI) [144].

These observations suggest that residual viral replication continues on ART in some patients, leading to the development of drug-resistance mutations and, as a consequence, therapy failure. However, most patients treated with modern ART do not demonstrate therapy failure. Therefore, as a next step, we studied the influence of decreased ART pressure (as a result of modestly decreased adherence to therapy) on the levels of cell-associated virological markers in patients on ART with long-term virological success. Surprisingly, we observed that even modest deviations from perfect adherence to ART (electronically measured adherence never fell below 70% in any patient), caused a significant longitudinal increase in the levels of usRNA, but no virological rebound in plasma [145]. As ART only blocks the infection of new cells, but not viral RNA transcription in cells infected before the start of therapy, the observed association of decreased ART pressure with increased usRNA levels in PBMC, in the absence of virological rebound in plasma, strongly suggests new replication cycles despite ART. Therefore, if an average ART-treated patient takes ~75% of the prescribed doses [146], our results suggest that most patients experience bursts of residual replication at some point during treatment. In most patients, these bursts of residual virus replication are probably self-limiting and therefore they do not lead to the development of drug resistance and therapy failure [90,147]. However, even if no ART failure is observed, low-level virus replication can trigger immune activation, with all the potentially morbid consequences thereof (see above).

Notwithstanding its importance, this residual virus replication, despite being readily detectable by quantifying CA HIV RNA, goes unnoticed in the clinic, where ART responses are monitored using commercial plasma viral load assays. This could simply be a sensitivity issue, i.e. ultrasensitive assays for plasma viremia [86] may also be able to detect these effects. Alternatively, CA viral RNA may be an intrinsically more suitable marker for monitoring therapy responses, *e.g.* because residual replication may occur by cell-to-cell transfer. By mathematical modeling and *in vitro* analysis, it was recently suggested that ART is much less efficient in preventing cell-to-cell HIV transfer that it is in stopping infection of cells by free virions [148]. If residual virus replication indeed occurs by cell-to-cell transfer in patients on ART, then CA HIV RNA is obviously a much more suitable biomarker to monitor this process than viral RNA in plasma.

Cell-to-cell HIV transfer is expected to preferentially occur in tissues (predominantly lymphoid tissues and gastrointestinal tract), where cell contacts are much more abundant, and where much higher viral DNA and CA RNA loads have consistently been found, than in peripheral blood [33,52,55,149-153]. Furthermore, three collaborating laboratories recently demonstrated both suboptimal drug penetration and high HIV levels in tissues compared to blood [154]. This finding might suggest that suppression of HIV replication in peripheral blood (as assessed by monitoring plasma viremia only) is “misleading” [154] in that it does not reflect ongoing viral replication in the tissues. Although it is clear that in order to get the full picture, one should look into tissues as well, it should be noted that unlike plasma viremia, CA HIV RNA level in PBMC is an excellent indicator of CA HIV RNA load in lymphoid tissue [55]. This can be due both to the constant trafficking of infected cells between tissues and peripheral blood and the extended half-life of infected cells compared to free virions, which can also be trapped in the follicular dendritic cell network [34]. In line with this reasoning, a recent study failed to show any compartmentalization of sequences derived from CA HIV RNA and DNA between the gut and peripheral blood [155], confirming the constant communication between these compartments [149].

Based on the evidence discussed above, we can speculate that monitoring ART response in peripheral blood may become less “misleading” if alternative HIV biomarkers are used for this purpose. Indeed, a number of studies recently attempted to demonstrate residual replication by observing the changes in plasma viremia, monitored with ultrasensitive assays, upon ART intensification with raltegravir [74,156,157], but no significant effects on plasma viremia were reported. In contrast, two reports documented a decrease in CA HIV RNA and a transient increase in 2-LTR circles (a form of episomal HIV DNA) upon the same intervention [143,158]. Notably, the two latter studies also failed to detect any effect of raltegravir intensification on plasma viremia. This difference may be partly explained by the fact that cell-associated HIV biomarkers more directly reflect virus replication, whereas plasma viremia is dependent not only on virus production but also on virus clearance. It remains to be established whether virus clearance is indeed more efficient and the half-life of free virions is indeed much shorter in patients on ART than in untreated patients (see above). However, if this is the case, then it may explain why it proved impossible to detect any (presumably small) decrease in virus production upon therapy intensification by measuring plasma viremia only, at least with infrequent sampling. Likewise, it could explain our observations of an increased CA HIV RNA level in suboptimal adherers in the absence of any virological rebound in plasma [145]. Therefore, even when cell-to-cell virus transfer is not taken into consideration, cell-associated viral markers may be more appropriate for monitoring ART responses than plasma viremia.

Besides CA HIV RNA, episomal viral DNA (e.g. in the form of 2-LTR circles) has been suggested as a biomarker of residual replication under ART [159]. This was based on the observed labile nature of 2-LTR circles, which thus could signify recently infected cells [159,160]. However, other studies failed to confirm the labile nature of 2-LTR circles [110,161,162]. Remarkably, transient accumulation of 2-LTR circles was demonstrated upon ART intensification with raltegravir, strongly suggesting that these episomal DNA molecules were derived from cells that had been recently infected despite ART [158]. Raltegravir treatment also reduced T-cell activation, which was higher at baseline in subjects with detectable 2-LTR circles [158,163]. However, these effects could not be reproduced by others, possibly due to the differences in sampling times or other methodological variations [164,165]. More recently, sequences derived from episomal HIV DNA but not from proviral DNA in patients on ART just prior to therapy interruption were shown to match those in rebounding virus after therapy interruption [166], emphasizing the dynamic nature of this marker. However, an earlier study failed to detect any significant shifts in the levels of 2-LTR circles during 2 weeks of structured treatment interruption, whereas clear shifts in CA usRNA were observed, suggesting that usRNA is a more dynamic and sensitive marker of HIV replication than 2-LTR circles [110].

When it comes to monitoring ART responses, one marker should not necessarily exclude the others. Both CA HIV RNA and episomal viral DNA have been linked to residual virus replication; therefore, if these two biomarkers are measured in parallel in a patient on ART, there is a better chance of timely identifying future ART complications (e.g. therapy failure due to suboptimal adherence) and indicating a clinical or behavioral intervention. In general, monitoring several biomarkers in parallel with sensitive assays, compared to the current clinical practice of relying on one virological marker only, which is measured with suboptimal sensitivity, would allow much better understanding, and ultimately control, of viral persistence in patients on therapy.

### Potential use of CA HIV RNA assays for HIV eradication strategies

The “gold standard” in measurement of replication-competent HIV reservoir is the quantitative coculture assay that determines an IUPM value [5]. However, this assay is labor-intensive, time-consuming, expensive, and requires large blood volumes (frequently obtainable only by leukapheresis). Therefore, alternative assays are urgently needed to support large-scale clinical trials exploring the effectiveness of HIV eradication strategies [5-8]. Several relatively easy-to-perform PCR-based assays have been developed for different HIV biomarkers, but it is still unclear which biomarker/assay can serve as a reliable surrogate for IUPM. Measurement of integrated DNA will not distinguish replication-defective from replication-competent virus, and quantitation of CA RNA (the “active HIV reservoir”) will miss cells that harbor transcriptionally silent proviruses but that can be activated to produce infectious virus (the “activatable HIV reservoir”). In any case, bulk assays will probably be of limited value to quantify frequencies of cells harboring replication-competent HIV: such a “magic bullet” assay should be performed at the limiting dilution or single-cell level.

One of the primary HIV eradication strategies that is currently being tested in clinical trials is reactivation of the latent viral reservoir by inducing virus production from latently infected cells using e.g. histone deacetylase inhibitors (HDACi) or other agents [5-7]. *In vitro*, the potency of these agents can easily be screened, but to test the efficacy of such compounds *in vivo* (or *ex vivo*), an assay should be used that measures reactivation of virus production in patients on ART. In this respect, it is important to determine which viral biomarker most adequately reflects virus production. As discussed above, plasma viremia reflects a balance between virus production and clearance (and the latter may depend on the viral load), some CA RNA^+^ cells may not produce virus, and the extracellular fraction of CA RNA does not necessarily originate from the attached cell. Still, in a recently published study that demonstrated the disruption of latency by vorinostat (an HDACi) in resting CD4^+^ T cells of patients on ART, CA HIV usRNA was used as an outcome measure [167]. Remarkably, in each of the eight participants, vorinostat caused an increase in both biomarkers of cellular acetylation and HIV usRNA [167], suggesting the usefulness of CA HIV RNA for monitoring the effectiveness of virus eradication strategies.

## Conclusions

Ultrasensitive measurement of plasma viremia has provided many important insights into HIV persistence during ART. Yet, to fully characterize the dynamics of viral reservoirs in patients on ART, sensitive and precise assays to quantify cell-associated HIV biomarkers are urgently needed. The observations discussed in this review suggest that CA HIV RNA is a promising candidate for the role of an alternative biomarker to be used in monitoring the virological response to ART and to novel HIV eradication strategies. However, more research is necessary to confirm these exciting observations. In particular, assay standardization is warranted, as the reproducibility across multiple laboratories is unknown. Therefore, comparative studies must be conducted to identify the most robust CA HIV RNA assay. In the ideal situation, such an assay should be HIV subtype-independent and capable of quantitation of CA viral RNA expression at the single-cell level. Further studies are also warranted in order to establish whether CA HIV RNA can be used as a reliable biomarker of persistent low-level virus replication/production in patients on suppressive ART. Furthermore, monitoring several biomarkers in parallel should provide advantages by increasing the assay robustness and sensitivity. In summary, novel, dynamic, viral biomarkers have to be characterized and assays to quantify them have to be developed, if our understanding of the virological processes in patients on ART is once to be taken “beyond undetectability”.

## Competing interests

The authors declare that they have no competing interests.

## Authors’ contributions

AOP wrote the manuscript. BB and VVL modified parts of the manuscript. All authors read and approved the final manuscript.
